# The ameliorative effect of atorvastatin on serum testosterone and testicular oxidant/antioxidant system of HFD-fed male albino rats

**DOI:** 10.12688/f1000research.25926.1

**Published:** 2020-11-05

**Authors:** Mohammed Esmail, Mohammed Kandeil, Ali Mahmoud El-Zanaty, Mohammed Abdel-Gabbar

**Affiliations:** 1 Biochemistry Department, Faculty of Science, Beni-Suef University, P.O. Box 62521, Beni-Suef, Egypt; 2 Biochemistry Department, Faculty of Veterinary Medicine, Beni-Suef University, P.O. Box 62511, Beni-Suef, Egypt; 3 Chemistry Department, Faculty of Science, Beni-Suef University, P.O. Box 62521, Beni-Suef, Egypt

**Keywords:** HFD, atorvastatin, atherogenic index, testosterone, antioxidants, MDA, testis, male rats

## Abstract

**Background**: There is a mutual effect between central obesity and low total serum testosterone. Moreover, oxidative stress acts as a bridge between obesity and its complications. Taken together, we aimed to evaluate whether atorvastatin (AS), a cholesterol-lowering drug, has protective potential against high fat diet (HFD)-induced low fertility, which was exemplified in serum testosterone determination. Moreover, we aimed to deduce a putative mechanism of action through evaluation of the testicular oxidant/antioxidant system.

**Methods**: Adult male albino Wistar rats ( *Rattus norvegicus albinus*) were divided into three groups: 1) normal control group, rats were fed a normal diet for four weeks; 2) HFD group, rats were fed an HFD for four weeks; and 3) AS group, rats were fed an HFD and 5 mg/kg/day atorvastatin for the last two weeks of the experiment. Serum atherogenic index, testosterone, and thyroid stimulating hormone were estimated. Moreover, testicular reduced glutathione and malondialdehyde contents, as well as glutathione-S-transferase, superoxide dismutase, and glutathione reductase activities were also determined. The statistical differences were analyzed using analysis of variance (ANOVA).

**Results**: AS ameliorated the increased level of serum atherogenic index induced by an HFD, as well as testicular malonaldehyde and reduced glutathione levels. On the other hand, AS increased the depleted level and activity of serum testosterone and testicular glutathione reductase, respectively, induced by HFD.

**Conclusion:** The ameliorative effect of AS on the deteriorated level of total serum testosterone induced by HFD might partially be due to oxidant/antioxidant disturbance. Further studies should be carried out to evaluate mTOR pathway contribution, which could enable researchers to deduce drugs targeting members of the oxidant/antioxidant system and/or mTOR pathway to ameliorate putative HFD-induced low fertility.

## Abbreviations

AX, atherogenic index; HDL-c, cholesterol of high-density lipoprotein; AS, atorvastatin; HFD, high fat diet; CN, normal control group; MDA, malondialdehyde; ELFA, enzyme-linked fluorescent assay; ROS, reactive oxygen species; GSH, reduced form of glutathione; SOD, superoxide dismutase; GST, glutathione-S transferase; TG, triacylglycerol; GR, glutathione reductase; TSH, thyroid stimulating hormone

## Introduction

Obesity or overweight is related to an elevated risk of many medical problems such as depression and infertility. In those that are obese or overweight, hyperlipidemia can increase the cholesterol content of platelets, endothelial cells, smooth muscle cells, neutrophils and polymorphonuclear leucocytes and may be a source of reactive oxygen species (ROS) ^1^. Moreover, increased free fatty acidemia and triglyceridemia, which are consequences of high fat diet (HFD) supplementation, are implicated in obesity complications ^2, 3^.

The WHO has reported that approximately 1.9 billion people are overweight and 650 million people are obese worldwide ^4^. Moreover, defective sperm quality constitutes 30% of infertile couples, who represent around 16% of the worldwide population ^5^. Interestingly, no precise cause can be elucidated for primary or secondary infertility in around 25% of couples ^6^. Although literature connecting semen parameters and obesity are contradictory, it is thought that there is a mutual effect between low serum testosterone and central obesity ^7^. It has been reported that an increased glycerol level, which is HFD-induced, might lead to a leaky blood-testis barrier, disrupting the homeostasis of the tubular fluid and promoting the apoptosis of germ cells ^8^.

 It has also been suggested that oxidative stress acts as a bridge between obesity and its complications ^9^. Dyslipidemia-generated elevated ROS and/or inflammatory substances are considered to be causes of metabolic disorders such as infertility ^10, 11^. High ROS levels may render testicular cells ineffective at maintaining a convenient environment for spermatogenesis ^12^. Hence, decreasing dyslipidemia may improve HFD-induced poor fertility and atorvastatin (AS), which is a cholesterol-lowering drug, may be beneficial in this way.

## Methods

### Ethical statement

All animal handling was in accordance with the instructions and guidelines of the Experimental Animal Ethics Committee, Faculty of Science, Beni-Suef University, Egypt (Approval Number: BSU/FS/2020/18).

### Experimental animals

A total of 30 apparently healthy adult male albino Wistar rats that initially weighed approximately 85 g were used in the beginning of this study to overcome any sudden death or diseases to rats. Later, we excluded seven rats because they did not achieve serum atherogenic index (AX) > 0.24, required for inclusion. Two rats died after sub-allocation of HFD-fed rats. Table 1 shows the final number of rats used and their allocation. The animals were purchased from Helwan University, Cairo, Egypt and then housed in the Biochemistry Department, Faculty of Science at Beni-Suef University. The rats were acclimatized for one week to exclude any intercurrent infection. Rats were kept in polypropylene cages with well aerated stainless-steel covers in a controlled environment that was maintained under a 12-hour light/dark cycle, a temperature of 24°C (±3°C) and 50–70% humidity. The rats were supplied with a standard diet and had free access to tap water. All efforts were made to reduce the number and suffering of the rats under investigation. These efforts included: 1) continuous checking of animal house air conditioning and continuous and careful supply of drinking water; 2) refreshing the animal house with ordinary air daily for 30 mins; 3) cleaning the cages and the animal house; 4) using the effective anesthesia dose; 5) carrying out euthanasia of animals far away from the other alive rats; 6) carefully disposing of euthanized animals far away from the other alive rats.

**Table 1.** The number of rats per group.

Sample description		
Group	Frequency	Percent
**CN**	7	33
**HFD**	9	43
**AS**	5	24
**Total**	21	100.0

CN, normal control; HFD, high fat diet; AS, atorvastatin.

### Materials

Normal standard food was purchased from Mecca Factory, Beni-Suef, Egypt. The high fat diet consists of the normal rat chow mixed with 15% palm oil and 1% cholesterol in accordance with Matos *et al.*, with some modifications ^13^. Compositions of normal standard and high fat foods are illustrated in Table 2. AS was purchased from Egyptian International Pharmaceutical Industries Co (EIPCO) 10th of Ramadan City, Egypt. All other chemicals were of analytical grade and were obtained from standard commercial suppliers.

**Table 2.** Composition of diet /100 g diet.

Composition	Normal chow diet	HFD
Yellow corn	60 %	50.5 %
Soybean	21 %	17.3 %
Fibers	11 %	9.4 %
Mineral mixture	4 %	3.6 %
Corn gluten	2 %	1.4 %
Limestone	1.5 %	1.3 %
NaCl	0.5 %	0.5 %
Cholesterol	0 %	1 %
Palm oil	0 %	15%

### Animal grouping and experimental procedure

Rats were divided into three groups as shown in Table 1. Selection of animals was carried out haphazardly without performing any prior randomization method.

1. Normal control group (CN): Rats were fed a normal diet, ad libitum, for four weeks; the whole period of the experiment. Rats were also given the equivalent volume of corn oil, as a vehicle, by oral gavage daily for the last two weeks.

2. HFD group: Rats were fed an HFD, ad libitum, for four weeks of the experiment, in addition to equivalent volume of corn oil by oral gavage daily for the last two weeks.

3. AS group: Rats in this group were fed a high fat diet for four weeks and treated orally with a dose of 5 mg/kg/day atorvastatin suspended in corn oil for the last two weeks of the experiment. The dose was double of that used by Oktem *et al.* ^14^.

The 30 rats were allocated to six cages; five rats/each cage. On the first day, we selected a cage for commencing treatment randomly, then, every day we selected the starting cage serially. Selection of an animal inside each cage was arbitrary. The oral gavage started at 10 AM until nearly 12 AM. Rats treatments were carried out at animal house in Faculty of Science. The route of AS administration was by oral gavage, which is easy and suitable for rats.

The first author was aware of most stages of the experiments. Most of the authors and technicians contributed to animal euthanasia and collection of specimens. Therefore, blinding was not achieved during allocation nor treating stages; however, it was achieved during assessment and data analysis stages.

The dose was adjusted every week based on the changes in body weight (b. w.) to ensure a constant dose per kg b. w. of rats over the entire period of study. After two weeks of the onset of the experiment, rats were fasted overnight (10–12 h) and blood samples were collected from the lateral tail vein for screening of the AX according to the formula: log10 (TG/HDL-c). Rats that exhibited AX less than 0.24 were excluded. This criterion was defined before any data was collected. Rats with AX more than 0.24 were allocated to the HFD group and AS-administered group for an extra two weeks. At the end of the experiment, the rats were deprived of food overnight and anaesthetized by inhalation with diethyl ether. Blood samples were collected from the jugular vein and then the rats were killed by sudden cervical decapitation while they were anaesthetized.

### Tissue sampling for biochemical tests

Immediately after weighing the testes, each testis was homogenized in 5 ml cold buffer (100mM potassium phosphate, PH 7.0, containing 2mM EDTA) per gram of tissue. For the biochemical analysis of antioxidants, including reduced glutathione (GSH) levels were determined according to the method of Beutler *et al.* ^15^. Moreover, glutathione-S-transferase (GST), superoxide dismutase (SOD), and glutathione reductase (GR) activities were also determined ^16– 18^. Besides that, determination of malondialdehyde (MDA) level, as an indicator for lipid peroxidation, was also estimated according to Ohkawa *et al.* ^19^.

***Determination of testicular GSH content.*** The protein content of the testicular sample was precipitated by adding an equal volume of 4 % sulphosalicylic acid. After centrifugation, 0.5 ml of the supernatant was added to 4.5 ml of 0.1 mM Bis-(3-carboxy-4-nitrophenyl)-disulfide reagent in 0.1 M phosphate buffer, pH 8. The color intensity was determined spectrophotometrically at 412 nm.

***Determination of testicular GST activity.*** After addition of glutathione (0.5 mM), and sodium phosphate buffer (0.1 M), pH 7.3, to the tissue sample, preincubation was carried out for 5 min at 37°C. Then, 1-chloro-2,4-dinitrobenzene (CDNB, 0.5 mM) was added to the incubation mixture. Thereafter, trichloroacetic acid solution (33%) was added and the mixture was centrifuged. The CDNB conjugate was measured in the supernatant at 340 nm. The quantity of enzyme that enhanced the formation of 1µmol of CDNB conjugate/mg protein/min was considered as one unit of enzyme activity. The molar extinction coefficient was 9.6 mM−1 cm−1.

***Determination of testicular SOD activity.*** The reaction mixture contained the sample, tris/EDTA buffer, 12.1%, pH 8, and pyrogallol 10 mM. SOD prevents pyrogallol auto-oxidation by removing superoxide anions. As described, 0.1 buffer was mixed with 1 ml tissue sample, then 0.05 ml pyrogallol was added. The absorbency was read at 0 and 10 mins after the addition of pyrogallol. One unit of enzyme activity is the amount of enzyme that resist a 50% change in extinction per one min. The modification used to determine SOD activity was the use of pyrogallol instead of cytochrome c. ΔA/min was calculated as follows:

Enzyme activity (U/ml) =

[ΔA/min (sample)]/ [ΔA/min (control) × 50 %]

Then U/ml was converted to U/g tissue.

***Determination of testicular MDA content.*** 1 ml of tissue homogenate was mixed with 2 ml of trichloroacetic acid (TCA, 7.5%) and then centrifuged. 2 ml of the formed supernatant was mixed with thiobarpeturic acid (TBA, 0.7 %) and boiled for 10 mins. The color intensity of the reactants (TBARS) was measured at 532 nm. An extinction coefficient of 156000 M−1 cm−1 was used for calculation.

### Biochemical assays

Serum triglyceride levels were determined according to the method of Tietz ^20^. Total serum cholesterol and HDL-cholesterol concentrations were detected according to the procedure of Allain *et al.* ^21^. Serum AX was calculated according to the equation [AX= Log(TG/HDL-C)] ^22^. AX is a useful measure of response to pharmacological intervention. Total serum testosterone and thyroid stimulating hormone (TSH) were estimated by enzyme-linked fluorescent assay (ELFA).

***Determination of serum triglyceride concentration.*** Serum triglyceride concentration was measured using the Randox triglycerides assay (Cat No. TR 213). The kit included reagent 2 which contained 4-chlorophenol (6 mmol/l), magnesium acetate (5 mmol/l), and tris buffer (50 mmol/l). Reagent 3 contained 4-amino antipyrine (1.0 mmol/l), ATP (1.0 mmol/l), lipases (≥100 U/ml), glyceolkinase (≥120 U/l), glycerol-3-phosphate oxidase (≥2.5 U/ml), and peroxidase (≥4.0 U/ml). Moreover, the working solution was performed by mixing the contents of reagent 3 with 30 ml of reagent 2. On the other hand, reagent 1 contained triglyceride (200 mg/dl) as a standard. As described, 10 μl of the serum or standard was added to 1 ml of the working reagent and then incubated for 5 min at 37°C. Thereafter, the absorbance of sample (Asample) and standard (Astandard) against reagent blank were measured at 456 nm. Triglycerides level (mg/dl) = (Asample/Astandard) × 200.

***Determination of serum cholesterol concentration.*** Serum cholesterol concentration was measured using the Randox Cholesterol assay (Cat No. CH 201). The cholesterol kit included reagent 1 which contained cholesterol (200 mg/dl) as a standard, while reagent 2 contained 3,5dichloro-2-hydroxybenzene sulfonic acid (4mmol/l), and tris buffer (50mmol/l) detergent (0.2 %). Reagent 3 contained cholesterol esterase (≥160 U/l), cholesterol oxidase (≥ 120 U/l), peroxidase (≥2000 U/l), and 4-aminoantipyrine (0.45 mmol/l). The working solution was prepared by mixing reagent 3 with 30 ml of reagent 2. The procedure for determining of serum total cholesterol level was similar to that of triglyceride determination, except the absorbance measurement was at 500 nm.

***Determination of serum HDL-cholesterol concentration.*** Serum HDL-cholesterol concentration was measured using the Randox HDL-cholesterol assay (Cat No. CH 203). Briefly, 50 μl of phosphtungestic acid (13.9 mol/l) was added to 500 μl of sample to precipitate LDL, VLDL and chylomicron fractions in the presence of magnesium ions, allowed to stand for 10 min, centrifuged for 10 min at 4000 rpm. Then, the supernatant was collected to determine the cholesterol content as described before. HDL-cholesterol concentration was calculated as follows:

HDL-cholesterol concentration (mg/dl) = A _sample_ × 180.

***Determination of serum total testosterone level.*** Serum total testosterone level was measured using the VIDAS® Testosterone II kit (Cat No. 414320, bioMérieux, France). The antibodies used were monoclonal anti-testosterone antibodies produced in mice. All assay steps were performed automatically by the instrument. The strip consisted of 10 wells covered with a labeled foil seal. The last well of each strip was a cuvette in which the fluorometric reading was performed. The wells in the center section of the strip contained the various reagents required for the assay.

The wells reagents included: well-1 (the sample well), well-2 (Conjugate): phosphate buffer, bovine albumin, alkaline phosphatase-labeled anti-testosterone antibody, and preservative (300 μL), and well-3 (Pre-treatment solution): phosphate buffer, bovine albumin, dissociation agent, preservative (600 μL). Additionally, wells-4, -5, and -6 were empty, while wells-7, -8, and -9 were (wash buffer) contained tris, surfactant, and preservative (600 μL). Well-10 (reading cuvette with substrate) contained 4-Methyl-umbelliferyl-phosphate (0.6 mmol/L), diethanolamine (DEA, 0.62 mol/L or 6.6%, pH 9.2, and 1 g/L sodium azide 300 μL).

Briefly, the procedure aimed, firstly, to isolate testosterone from the carrier proteins in the sample by using the pre-treatment solution. The pre-treated sample was transferred to a well containing a testosterone antibody (conjugate) labelled with alkaline phosphatase. For the anti-testosterone-specific antibody sites, the antigen in the sample and the testosterone antigen attached to the SPR ® inner wall. During the washing steps, unbound pieces were removed. The substrate (4-Methylumbelliferyl phosphate) was cycled in and out of the SPR ® during the final detection stage. The hydrolysis of this substrate was catalyzed by the conjugate enzyme into a fluorescent product (4-Methylumbelliferone), whose fluorescence was measured at 450 nm. The fluorescence intensity was inversely proportional to the testosterone concentration present in the sample. The results were automatically determined by the instrument at the end of the assay with regard to the calibration curve stored in memory.

***Determination of serum TSH level.*** Serum TSH level was measured using the VIDAS® TSH kit (Cat No. 30400, bioMérieux, France). The protocol was like that of total testosterone determination except we used mouse monoclonal anti-TSH antibodies instead of anti-testosterone antibodies.

### Statistical analysis

Data are expressed as mean ± SE. The statistical differences between continuous variables was analyzed using analysis of variance (ANOVA). Statistical analysis was performed using SPSS v.24 (SPSS Inc., released 2007, SPSS for Windows, version 16.0, Chicago, IL). Values with *p* < 0.05 were considered significant.

## Results

Data resulting from our experiment were deposited in Harvard Dataverse ^23^. Supplementing rats with an HFD produced a significant increase ( *p* < 0.05) of 31% in serum AX relative to CN. However, administering AS ameliorated this effect by significantly decreasing serum AX by 18% relative to the HFD-fed group. HFD caused a profound decrease of 62% in total serum testosterone, while AS alleviated this negative effect by increasing the total serum testosterone level by 92% ( *p* < 0.05). However, neither HFD nor AS showed a significant effect on serum TSH at *p* ≥ 0.05 ( Table 3).

**Table 3.** Changes in serum levels of total testosterone (T. T.; ng/ml), thyroid stimulating hormone (TSH; ng/ml) and atherogenic ratio (AX) as a result of supplementation of AS to HFD-fed rats.

	**T. T.**	% change	**TSH**	% change	**AX**	% change
**CN**	2.5±0.2 ^c^	-	1.16±0.37 ^a^	-	0.52±0.005 ^a^	-
**HFD**	0.96±0.04 ^a^	-62	1.08±0.21 ^a^	-7	0.68±0.03 ^b^	31
**AS**	1.84±0.06 ^b^	92	1.34±0.35 ^a^	24	0.56±0.009 ^a^	-18

Values are expressed as means ± SE. Means that have different superscript letters are significantly different at P ≤ 0.05. % changes were calculated by comparing HFD means with CN means and AS means with HFD means. CN, normal control; HFD, high fat diet; AS, atorvastatin.

Regarding MDA, HFD exerted a tremendous increase in testicular levels of MDA of 113%. AS exhibited an amelioration of this effect, causing a 54% decrease in MDA levels relative to the HFD group ( *p* < 0.05). Testicular GSH content was profoundly increased by HFD supplementation by 87%, while AS supplementation caused a 30% decrease in GSH content ( *p* < 0.05) ( Table 4).

**Table 4.** Changes in testicular levels of malondialdehyde (MDA; nmol/g.tissue) and reduced glutathione (GSH; mg/g.tissue) as a result of supplementation of AS to HFD-fed rats.

	**MDA**	% change	**GSH**	% change
**CN**	8.4±0.16 ^a^	-	5.96±0.2 ^a^	-
**HFD**	17.9±0.56 ^b^	113	11.14±0.17 ^c^	87
**AS**	8.3±0.29 ^a^	-54	7.84±0.13 ^b^	-30

Values are expressed as means ± SE. Means that have different superscript letters are significantly different at P ≤ 0.05. % changes were calculated by comparing HFD means with CN means and AS means with HFD means. CN, normal control; HFD, high fat diet; AS, atorvastatin.

GR activity was decreased by 42% by HFD but increased by 112% by AS ( *p* < 0.05). There were moderate increases of 14% and 11% in SOD and GST activities, respectively, after HFD supplementation. However, a decrease of 4% was observed in SOD activity after AS administration ( *p* < 0.05), while AS produced an insignificant change in GST activity relative to the HFD group ( *p* ≥ 0.05) ( Table 5).

**Table 5.** Changes in testicular activities of glutathione reductase (GR; U/L), superoxide dismutase (SOD; U/g.tissue), and glutathione S-transferase (GST; U/g.tissue) as a result of supplementation of AS to HFD-fed rats.

	**GR**	% change	**SOD**	% change	**GST**	% change
**C.N**	229.4±1.7 ^b^	-	621.1±1.5 ^a^	-	9.29±0.25 ^a^	-
**HFD**	132.8±3.1 ^a^	- 42	709.1±4.2 ^c^	14	10.34±0.38 ^b^	+11
**AS**	281.2±16.3 ^c^	112	680±13.3 ^b^	- 4	10.26±0.16 ^b^	- 1

Values are expressed as means ± SE. Means that have different superscript letters are significantly different at P ≤ 0.05. % changes were calculated by comparing HFD means with CN means and AS means with HFD means. CN, normal control; HFD, high fat diet; AS, atorvastatin.

## Discussion

Lipid profile amelioration is considered to be an important target in order to foster β-oxidation of fatty acids, otherwise fatty acids would be oriented towards the esterification pathway, leading to accumulation of TAG and LDL cholesterol ^24^. Hence, statins such as AS can help in this trend. They can up-regulate the LDL receptor and reduce cholesterol absorption and biosynthesis, an effect that leads to rapid clearance of LDL particles from circulation ^25^.

Statins were thought to improve sperm parameters regarding dyslipidemia. However, Pons-Rejraji *et al*. counteracted this finding, as statins might induce local inflammation or oxidative stress, with persistent effects on prostatic and epididymal secretion ^26^. In agreement with this, some evidence has shown that statins might be detrimental to testosterone production ^27^ and might induce impotence ^28^. Other studies indicated that patients undergoing statin therapy to lower serum cholesterol levels have neither androgen deterioration ^29^ nor impotence ^30^ due to sterol regulatory element binding protein (SREBP)/Srd5a2 system alteration ^31^. The different pharmacokinetic or physicochemical properties of various statins may contribute considerably to total serum testosterone levels.

 To share in this debate, we introduced an HFD to rats and treated them with AS. In our experiment, an HFD profoundly decreased serum testosterone, which was counteracted by supplementing with AS, although serum levels were not elevated to that of the normal control. It was previously reported that the decrease of total serum testosterone in obese men is due to increased serum estrogen as a result of increased aromatase activity ^32^. 

The detrimental effects of HFD may be mediated, in our study, via an elevated testicular malondialdehyde level, which is a marker of oxidative stress. However, the ameliorative effect of AS may be partially explicated by lowering serum AX and testicular malondialdehyde levels. Surprisingly, HFD markedly elevated testicular glutathione level and GR activity, which may be a kind of compensatory mechanism. The substantial contribution of the oxidant/antioxidant system in the deterioration effects of HFD on fertility was obvious.

Additionally, cholesterol synthesis is not completely inhibited in peripheral tissues in statin-treated patients ^33^. Therefore, enough cholesterol is available in gonadal and adrenal cells to maintain steroid hormone synthesis; this in line with our data.

Accumulated data suggest mTOR as a promising mechanism for explaining HFD-caused detrimental effects on fertility. In one study, mTOR and p70s6k were weakly expressed in the HFD group, which may be reversed by statin ^34^. 

Our data enables researchers to deduce drugs targeting members of the oxidant/antioxidant system and/or mTOR pathway to ameliorate putative HFD-induced infertility.

## Data availability

### Underlying data

Harvard Dataverse: Replication Data "The Ameliorative Effect of Atorvastatin on Fertility and Testicular Oxidant/Antioxidant System of HFD-Fed Male Albino Rats". https://doi.org/10.7910/DVN/9LEX1O ^23^.

This project contains the following underlying data:

- Stat (AS).docx (statistics report of data)
- Outcomes (AS).docx (raw GSH, GR, SOD, and GST and MDA levels, as well as serum total testosterone and TSH levels for all 21 rats)

Data are available under the terms of the Creative Commons Zero "No rights reserved" data waiver  (CC0 1.0 Public domain dedication).
