# Supplementary material for: The ameliorative effect of atorvastatin on serum testosterone and testicular oxidant/antioxidant system of HFD-fed male albino rats
Source: F1000Res. 2020 Nov 5;9:1300. [Version 1] doi: 10.12688/f1000research.25926.1 (PMC7876586; doi:10.12688/f1000research.25926.1)
Supplement: Peer review history

# Peer review: Reviewer response for version 1

**DOI:** 10.5256/f1000research.28612.r77679

**Published:** 2021-02-10

**Authors:** Mostafa Waly

**Affiliations:** 1 Food Science and Nutrition Department, College of Agricultural and Marine Sciences, Sultan Qaboos University, Muscat, Oman

This is a well written article and the theme of the reported research will add needed knowledge in the field.

 I have few minor comments to optimize the quality of the manuscript as follow:

- Please provide the weight gain of the experimental animals throughout the duration of the experiment. I would recommend a figure as an illustration.
- The statistical analysis, One Way ANOVA, should be followed by post analysis; for example Tukey's test. This will provide significant power for the reported results.

Is the work clearly and accurately presented and does it cite the current literature?

Yes

If applicable, is the statistical analysis and its interpretation appropriate?

Yes

Are all the source data underlying the results available to ensure full reproducibility?

Yes

Is the study design appropriate and is the work technically sound?

Yes

Are the conclusions drawn adequately supported by the results?

Yes

Are sufficient details of methods and analysis provided to allow replication by others?

Yes

Reviewer Expertise:

Biochemistry and oxidative Stress

I confirm that I have read this submission and believe that I have an appropriate level of expertise to confirm that it is of an acceptable scientific standard.

# Peer review: Reviewer response for version 1

**DOI:** 10.5256/f1000research.28612.r75299

**Published:** 2021-01-25

**Authors:** Ahmed A. Mousa

**Affiliations:** 1 Department of Biochemistry and Chemistry of Nutrition, Faculty of Veterinary Medicine, University of Sadat City, Sadat City, Egypt

Authors have done a good effort in this work and they concluded that AS has ameliorative effect on the deteriorated level of total serum testosterone induced by HFD might partially be due to oxidant/antioxidant disturbance.

 Major comments:

- Would you have a reference for induction of obesity in just for 4 weeks with palm oil and cholesterol?
- What was the final weight of rats at the end of experiment?
- Why did authors not provide any study on assessment of sperm parameters?
- EIPCO Commercial HFD, what is the purpose for it in markets or is it only for research purposes? 
- Why did authors not formulate HFD by themselves?
- Please add name and model of spectrophotometer and ELISA plate reader.

Minor comment

- Add the full name of mTOR in abstract section and abbreviations section.

In Methods: delete **All efforts were made to reduce the number and suffering of the rats under investigation. These efforts included: 1) continuous checking of animal house air conditioning and continuous and careful supply of drinking water; 2) refreshing the animal house with ordinary air daily for 30 mins; 3) cleaning the cages and the animal house; 4) using the effective anesthesia dose; 5) carrying out euthanasia of animals far away from the other alive rats; 6) carefully disposing of euthanized animals far away from the other alive rats**.

 **The first author was aware of most stages of the experiments. Most of the authors and technicians contributed to animal euthanasia and collection of specimens. Therefore, blinding was not achieved during allocation nor treating stages; however, it was achieved during assessment and data analysis stages**.

Is the work clearly and accurately presented and does it cite the current literature?

Yes

If applicable, is the statistical analysis and its interpretation appropriate?

Yes

Are all the source data underlying the results available to ensure full reproducibility?

Yes

Is the study design appropriate and is the work technically sound?

Yes

Are the conclusions drawn adequately supported by the results?

Yes

Are sufficient details of methods and analysis provided to allow replication by others?

Yes

Reviewer Expertise:

Oxidative stress and andrology

I confirm that I have read this submission and believe that I have an appropriate level of expertise to confirm that it is of an acceptable scientific standard.
